# Dietary exposure to tetracycline residues through milk consumption in Iran

**DOI:** 10.1186/s40201-015-0235-6

**Published:** 2015-11-23

**Authors:** Fathollah Aalipour, Maryam Mirlohi, Mohammad Jalali, Leila Azadbakht

**Affiliations:** Food and Drug Administration, Sharekord University of Medical Sciences, Shahkord, Iran; Food Security Research Center, School of Nutrition and Food Sciences, Isfahan University of Medical Sciences, Hezargrib Street, Isfahan, Iran

**Keywords:** Risk assessment, Tetracycline, Milk, Exposure, Iran

## Abstract

**Background:**

The abundant use of tetracycline antibiotics in veterinary medicine may result in the presence of their residues in milk at unsafe concentrations that can adversely affect public health. The aim of the current study was to evaluate the risk of tetracycline residue (TET) intake via milk consumption amongst different age groups of human consumers in Iran.

**Methods:**

To quantify the drug residues, HPLC analysis was performed under isocratic conditions using UV detection at 355 nm. Milk consumption patterns were determined using a recent nutrition survey in Iran.

**Results:**

The average concentration of total TETs in milk was determined to be 252.41 μg/kg, which is approximately 2.5 times greater than the maximum residue limit (MRL) set by codex. Of the four different tetracycline antibiotics analyzed, oxytetracyclin had the highest share (86 %) of the determined contamination.

**Discussion:**

Daily exposure to TETs through milk using an average data on milk consumption was estimated to range from 58–62 μg. but, distribution based exposure to TETs in milk appeared as 0–99.3 μg per day.

**Conclusions:**

Risk characterization of dietary exposure to TETs residue via milk intake in different age groups showed that considering the standard dietary recommendation that advices on two servings of milk per day (480 ml), consumers may receive 7–30 % of the determined ADI via bovine milk consumption.

## Background

Tetracyclines (TETs) are a group of broad-spectrum antibacterial agents indicated for use against a large variety of microorganisms including aerobic, anaerobic, Gram-positive and Gram-negative pathogenic bacteria, as well as some types of protozoa. They are widely used in veterinary medicine for prevention and treatment of infectious diseases and also serve as growth promoter additives in animal feed [1]. Although the use of TETs in the treatment of human infectious disease was more common in the past, they are still routinely used, especially in patients susceptible to allergic reactions to ß-lactams and macrolides. Four types of tetracycline antibiotics are routinely used in animal husbandry practice: oxytetracycline (OXT), tetracycline (TET), chlortetracycline (CHT) and doxycyclin (DOT). In cattle, oxytetracycline is frequently applied to treat enteritis, endometritis, septicemia, and mastitis, as well as other bacterial infections [2]. They may be introduced orally through feed or drinking water, or parenterally via intra-mammary infusion [3]. In either case, upon administration, the drug is partially excreted through maternal milk. Its concentration in milk is approximately 70 % of that of the concentration within maternal serum [4–6]. Improper veterinary use of tetracyclines, as well as inadequate knowledge of the necessary withdrawal time, can easily make the tetracyclines or their derivatives appear in the marketed milk.

Intake of contaminated milk can result in adverse human health effects including allergic reactions, the development of bacterial resistance, and the risk of teratogenicity when administrated during the first trimester of pregnancy. Moreover, primary and permanent teeth discoloration often occurs when milk contaminated with tetracycline residues is consumed by infants or children less than 12 years old [7].

Regulations have been established by food safety authorities in order to prevent or reduce the negative health effects of tetracyclines on consumers; the Food and Agricultural Organization and World Health Organization (FAO/WHO) and the European Union (EU) have recommended a maximum residue limit (MRL) of 100 μg/kg for tetracycline, oxytetracycline and/or chlortetracycline (singly or in combination) in milk [8–10]. Meanwhile, the U.S. Food and Drug Administration (FDA) has set an upper legal level of 300 μg/kg for the combined residues of TET, OXT and CHT [11]. The Joint Expert Committee on Food Additives has recommended an acceptable daily intake (ADI) for TETs residues at a concentration of 0–30 μg/kg _bw_/day [8].

In Iran, several studies have been published examining the presence of antibiotic residues in milk. Chronologically, the levels of antibiotic residues in milk found to exceed recommended levels have increased from 5 % in the first study completed in 2009 to more than 20 % in the latest one in 2012 [12–15], indicating that in Iran the risk of antibiotic intake through milk consumption must be assessed using both qualitative and quantitative methods. In this regard, previously published information has been insufficient in determining the presence of antibiotic residues in milk as they have primarily focused on the general antibacterial activity, while determination of specific antibiotics was rarely carried out.

The objective of this study was to evaluate the risk associated with the presence of antibiotic residues in marketed milk in Iran. A chemical risk assessment is defined as the likelihood of a chemical having adverse health effects and is divided into four steps: 1) hazard identification; 2) hazard characterization/dose-response assessment; 3) exposure assessment; and 4) risk characterization [16]. In the first component of the present study, the utilization pattern of antibiotic drugs in livestock and poultry farms was surveyed. It was indicated that TETs, in particular oxytetracycline, were in abundant use on both animal and poultry farms [17]. Secondly, a market study was carried out and the contamination rate of pasteurized and sterilized milk (*n* = 180) from 25 commercial brands offered in retail was investigated. Approximately 20 % of the samples tested were found to have antibiotic residues at levels greater than the legally permitted levels [18].

In the current study, the analytical determination of four types of tetracyclines within the previously identified contaminated milk samples was planned, and then, based on the existing and representative data on the average milk consumption for adults and children, the risk of TETs intake via milk intake in Iran was evaluated.

## Method

### Milk samples

A total of 187 commercial cows' milk samples, consisting of 33 sterilized and 154 pasteurized products, from 24 Iranian dairy brands were randomly collected from the Shahre-kourd city market from January to July 2012. Sample size was determined based on the previously reported values of antibiotic contaminated milk samples in Iran (5–20 %) [12–15]. Also availability of the commercial brands in the market was considered in sampling. Samples were sent to the Food and Drink Quality Control laboratory of the Shahre-kourd University of Medical Science, and were examined for the presence of antimicrobials.

### Screening test

Screening tests were conducted using a commercial kit (Eclipse 100-kit (Zeu. Inmunotec, Spain)) by which the presence of antimicrobial residues could be detected based on the inhibition of microbial activity. According to the manufacturing company, the sensitivity of the test for TETs is between 50–150 μg/kg. Positively detected milk samples were stored at −20°c until HPLC analysis.

### Chemical and reagents

Analytical standards of tetracycline antibiotics, including tetracycline hydrochloride, oxytetracyclin hydrochloride, chlortetracyclin hydrochloride and doxycyclin hydrochloride, were provided from Alderck Company. Analytical grade chemicals including acetic acid, sodium chloride, oxalic acid, citric acid, ethylene-diaminetetraacetic acid (EDTA) and disodium hydrogen phosphate (Na2HPO4) were provided from Merck, Germany. Methanol and Acetonitril of HPLC grade were also from Merck-Germany. Solid phase extraction cartridges (500 mg, 6 ml) were provided from Macherey-Nagel Company (Germany).

### Preparation of standard curves

Stock solutions of tetracyclines in methanol (Merck-Germany) at the concentration of 100 μg/ml were prepared using pharmaceutical standards; working standards in methanol were made in the concentration rage of 50–400 ng/ml. The working standards were kept in refrigerator for less than one month [19].

### Preparation milk samples

Two milliliters of 20 % TCA (trichloroacetic acid-Merck-Germany) and 20 ml of EDTA-McIlvaine buffer were added to 5 ml of milk sample. EDTA-McIlvaine was prepared using 11.8 g of citric acid monohydrate, 13.72 g of disodium hydrogen phosphate dehydrate, and 33.62 g of ethylene-diaminetetraacetic acid disodium salt dissolved in 1 l of double-distilled water (0.01 M). The mixture was then mixed thoroughly and centrifuged at 4000 rpm for 20 min. The resultant supernatant was removed and then applied to the solid phase extraction (SPE HLB C18) cartridge that had been previously activated with 3 ml methanol followed by rinsing with 2 ml double-distilled water. After loading each sample, the cartridge was washed with 2 ml methanol solution (5 %) in double-distilled water, the analytics was eluted with 3 ml of pure methanol, instantly removed under a nitrogen stream, and the residue was resolved in 1 ml mobile phase. In each batch of injection, 10 μl of the given aliquot or standard was filtered through a 0.45-μm micro-filter and injected in to system [20].

### Instruments and HPLC condition

The UHPLC-KUNAER system was used (model A69420, Germany) which was equipped with double pumps (model A60015, Germany), UV-vis detector (model MW-1 A61031, Germany) set at 355 nm and auto sampler (model AS-1 A63500, Germany). Separation was carried out under isocratic conditions using a C_18_ Column, 250 mm × 4.6 mm I.D., containing 5 μm particles. The mobile phase consisted of 0.01 M oxalic acid solution, methanol and acetonitril (60:25:15 v/v/v), and was filtered through a 0.45-μm micro-filter at an adjusted flow rate of 1 ml/min.

### Validity parameters

Obtained validity parameters, including linearity, limit of detection (LOD), limit of qualification (LOQ), entry-day precision, and intra-day precision, are listed in Table 1. Calibration curves of mixed standard TETs were provided for five concentration levels (50, 100, 200, 300 and 400 ng/ml) in a blank milk sample.

**Table 1:** Validity parameters in analytical determination of tetracycline residues in milk

Parameter	Oxytetracyclin	Tetracycline	Chlortetracycline	Doxycycline
LOD ng/ml	1.18	1.12	1.7	1
LOQ ng/ml	4	4	5	3
Regression coefficient	0.991	0.994	0.966	0.915
Retention Time (min)	4.56	5.01	7.90	11.78
Inter day precision *N* = 3 day	0.78	1.06	3.09	8.07
Entry day precision	0.59	1.33	3.19	6.13
Linearity equation	Y = 0.001376x	Y = 0.0157643x	Y = 0.0308564x	Y = 0.019085x

The limit of detection (LOD) and the limit of quantification (LOQ) were determined based on signal to noise ratios. For the entry-day precision, the relative standard deviation (RSD) of four repeated measurements of a blank milk sample containing 500 ng/ml mix standard through a one-day experiment run was considered. For the determination of intra-day precision, the relative standard deviation of the repeated measurements of a milk sample containing 100 ng/ml standard mix in blank milk sample through four subsequent days was taken in to account. A recovery test was performed using the spiked blank milk in three concentration levels (100, 200, 300 ng/ml) of mix standard. The obtained recovery percentages are presented in Table 2. The highest and lowest recovery rates were obtained for tetracycline (88.78 %) and chlortetracyclin (64.68 %) respectively. Based on the European Commission, Regulation 2002/657/EC, the RSD should be lower than 15 % [21].

**Table 2:** Recovery (%) of tetracyclines spiked in different concentration levels

Spiked samples	Oxytetracyclin	Tetracycline	Chlortetracycline	Doxycyclin	Total
100 ng/ml	68.67 ± 8.6^a^	90.73 ± 3.4	69.1 ± 3.1	62.4 ± 10.4	72.73 ± 19.1
Precision	0.41^b^	0.69	5.29	19.04	1.02
200 ng/ml	63.67 ± 7.9	84.68 ± 2.3	59.76 ± 2.8	65.64 ± 3.1	67.99 ± 6.1
Precision	0.62	0.84	2.21`	2.34	0.16
300 ng/ml	68.14 ± 13.2	72.93 ± 11.2	65.17 ± 3.5	71.47 ± 5.9	69.16 ± 17.4
Precision	0.99	1.63	1.78	2.84	0.46
Mean	66.83 ± 9.9	82.78 ± 5.6	64.68 ± 3.1	66.52 ± 6.4	69.96 ± 14.2
Precision	0.78	1.06	3.09	8.07	0.67
*n* = 20					

^a^Means of recovery data ± standard deviation for 20 measurements

^b^Precision is expressed as the percentage of relative standard deviation (RSD)

### Exposure assessment of TETs residues in milk

Exposure assessment was performed using deterministic (point estimate) and probabilistic distribution-based or population-related) approaches for three target groups; infants, children and adults were included.

Per capita milk consumption was obtained from the official report released by the deputy head of Iran Dairy Industries Society (IDIS) [22] as 85–90 kg and used in the deterministic exposure assessment. The reported results of three recent Iranian nutrition surveys were used to derive the lowest, the highest and average milk intake among adults and children [23–25] in the probabilistic exposure estimation. The following equations were used to determine the risk associated with the TETs residue through milk intake:

1 $$ \mathrm{Intake}\ \mathrm{of}\ \mathrm{TETs}\ \mathrm{residue}\left(\upmu \mathrm{g}/\mathrm{day}\right) = \mathrm{Mean}\ \mathrm{residue}\ \mathrm{concentration}\left(\upmu \mathrm{g}/\mathrm{kg}\right) \times \mathrm{daily}\ \mathrm{milk}\ \mathrm{consumption}\left(\mathrm{kg}/\mathrm{day}\right) $$

2 $$ \%\mathrm{A}\mathrm{D}\mathrm{I} = 100 \times \mathrm{I}\mathrm{ntake}\ \left(\upmu \mathrm{g}/\mathrm{day}\right)\ /\left[\left(\mathrm{A}\mathrm{D}\mathrm{I}\ \left(\upmu \mathrm{g}/{\mathrm{kg}}_{\mathrm{bw}}/\mathrm{day}\right) \times \mathrm{body}\ \mathrm{weight}\ \left(\mathrm{kg}\right)\right]\right. $$

### Statistical analysis

The Statistical Package for Social Science (SPSS) software Version-19 was used for data analysis using descriptive statistics.

## Results

The screening test revealed that 19.78 % of the samples were positive for antibacterial residues at levels above the defined MRL. Twenty-eight of the total 178 milk samples (14.97 %) were detected to have residues below the regulatory limit and 122 milk samples negatively responded to the test.

The results of the HPLC determination of TETs residues in the positive samples are presented in Table 3. Amongst the four investigated types of TETs, oxytetracycline distribution frequency and concentration levels were most prevalent, as it was found to be present in all of the tested samples (*n* = 37) and that it covered 86.71 % of the total concentration of measured TET residues. Considering that the standard limit of TET residues in milk defined by MRL-EU is 100 μg/kg [9, 10], the average concentration of the four examined drugs among the positive samples (1090 μg/kg) and in the whole sample population (252.4 μg/kg) were approximately 11 and 2.5 times greater than the standard level, respectively. Oxytetracycline in particular had the highest share in this contamination and its mean concentrations in the positive and total milk samples were 30 and 7.2 times greater than the mentioned permitted limit, respectively. The other TET drug types were detected in low frequency; chlortetracycline and doxycycline residues were found in just five and two out of the 37 samples. Their mean values were also measured at concentrations below the legislated level.

**Table 3:** Tetracycline residues in commercial milk samples(μg/kg)

	Number of positive samples	Minimum	Maximum	Mean^a^	Each drug ratio to the total TETs concentration
Oxytetracyclin	37	197.65	2137.27	945.90	86.71
Tetracycline	2	<LOD	241.45	28.71	2.63
Chlortetracycline	5	<LOD	288.19	71.93	6.59
Doxycycline	2	<LOD	206.13	44.283	4.05
Total residues in positive samples	37	220.93	2452.06	1090.83	100
Total residues in total samples	187			252.41^b^	

^a^The average concentration in the positive samples

^b^Mean concentration of TET residue in the whole sampled population was calculated as an average of 1090.83 μg/kg for positive samples (20 % of the total share), 100 μg/kg (maximum residue level) for 15 % of the total share and 25 μg/kg for the rest (65 %) which appeared negative in the screening test

Using a deterministic approach and considering 233–246 g milk intake per day, the daily intake of TETs residues through long term milk consumption by every Iranian was roughly estimated to be 58–62 μg. While, probabilistic exposure estimation using cross sectional studies revealed that such an intake could be between 8.5–99.3 μg for adults (more than 18 years old) and 0–28 μg for children under 12 years old during a short term exposure. For younger children and infants, since exact data on regular cows’ milk consumption was not available, frequency distributed exposure could not be estimated.

In Table 4, the result of risk characterization of TETs residue via milk consumption is presented. Estimated daily exposure data for adults and children is also depicted in this table. The highest level of acceptable daily intake (ADI) of TETs established by JECFA (30 μg/kg _bw_ /day) was taken into account for risk characterization.

**Table 4:** Risk characterization of dietary exposure to tetracycline residues^a^ via milk intake in different age groups

Age group	Body weight (Kg)^b^	Probabilistic						Deterministic	
		Low consumers^c^		Average Consumers^d^		High Consumers^e^		Per capita consumption^f^	
		EDI^g^ (μg/Kg_bw/d_	%ADI	EDI (μg/Kg_bw/d_	%ADI	EDI (μg/Kg_bw/d_	%ADI	EDI (μg/Kg_bw/d_	%ADI
Adults (>18y)	60	0.141	0.47	0.7	2.3	1.65	5.5	0.96	3.2
Children (6 < y < 12)	30	-	-	0.44	1.46	0.92	3.16	4.13	13.7
Children (2 < y < 3)	13	ND	ND	ND	ND	ND	ND	4.43	14.7

^a^The average concentration of tetracycline in the total milk samples (252.4 μg/kg) was taken in to consideration in all cases

^b^Body weights for different age groups were derived from FAO/WHO guideline and relevant nutritional studies

^c^The lowest amount of milk intake among Iranian adults and children were adapted from [24] as 33.6 ml/day and [25] as negligible, respectively

^d^The average amount of milk intake among Iranian adults and children were adapted from [23] as 165 ml/day and [25] as 52 ml/day, respectively

^e^The highest amount of milk intake among Iranian adults and children were obtained from [24] as 393 ml/day and [25] as 113 ml/day, respectively

^f^Per capita milk consumption data is considered as 246 g/d (the upper side of the rage announced by IDIS)

^g^Estimated daily intake (EDI):[Milk consumption X 252.41(mean concentration of TETs in milk)/1000]/body weight

Based on the presented results, estimated daily exposure for the high-intake consumers could reach up to about 6 % of the defined ADI at short term exposure. While, considering per capita milk consumption in long time, children are exposed to more possible risk due to their less body weight.

## Discussion

Today, in addition to the adverse effects that can occur as a result of the use of veterinary drugs, antibiotic resistance is considered to be a major threat to human health. The presence of antibiotic residues in foods of animal origin have attracted attentions as to whether their long-term intake could be contributing to the development of antibiotic resistance in humans. The present study demonstrated that tetracycline residues in the commercial milk brands marketed in Iran can easily exceed the safety limits recommended by the EU. The average concentration of oxytetracycline, tetracycline, chlortetracycline and, doxycycline residues in the milk samples studied (*n* = 187) were found to be 218.86, 6.63, 16.66, and 10.22 μg/kg, respectively. However, all of the tested samples in the present study were compliant with the standards set by the FDA as the sum of concentration of tetracycline, oxytetracycline, chlortetracycline and doxycycline were less than the MRL set by FDA (300 μg/kg). This difference may be in part due to the different dairy consumption patterns in the US and European zones versus those in Iran, and could be indicative of the necessity to establish national standards based on the dietary intake patterns in Iran.

Recently, several descriptive studies in Iran have been carried out on the presence of tetracycline residues in milk. However, the reported contamination levels were less than those found in the current study. In one of the previous studies which examined 90 pasteurized milk samples, the researchers showed that 7.8 % of the tested samples were contaminated with oxytetracycline and tetracycline residues at concentrations far below the safe limit [26]. Another study investigated the residue levels of three drugs of the tetracycline family including tetracycline, oxytetracycline and chlortetracycline (TCs) in different types of thermally-processed bovine milk collected from Ardebil. The violation rate of 24.4 %, 30 % and 28.6 % for pasteurized, sterilized and raw milk samples were reported respectively, while the overall concentration of tested TETs in milk samples averaged as 97.6 μg/kg [27]. In a similar study conducted in the U.S, on average, 62.2 μg/kg of TETs were identified in the marketed milk samples, in which 57.2 % of the total detected contamination was due to oxytetracycline [5]. In another study carried out in Ethiopia, each kilogram of raw marketed milk was revealed to contain 142 μg of oxytetracycline residue [28]. In Yugoslavia, Vragović et al. reported that, on average, the examined milk samples were contaminated with 1.5 μg/kg of tetracycline [29]. This was significantly lower than the levels identified in the present study (6.63 μg/kg). This suggests that tetracycline-derivative residues in milk in Iran are more controversial than in the aforementioned countries. However, in a report from Romania, TET contamination of milk averaged 272.5 μg/kg, which is comparable to the results of the current study [30]. Beta-lactam drugs such as penicillin were previously found as a commonly consumed veterinary antibiotic through food in US [31].

In the present study, with respect to the exposure estimation, for children group, the deterministic approach resulted in a higher estimated daily exposure to TETs than the probabilistic model. This could be due to the low level of milk intake in a group of children studied in the used nutritional survey. Although none of the investigated groups in the present study received the TETs residues at a level greater than the higher board of the ADI (30 μg/kg _bw_/day), considering the standard dietary recommendation that advise on 2 servings of milk per day (480 ml), consumers may get 7–30 % of the determined ADI via bovine milk consumption. The estimated risks for children passing their infancy could be several times more than the average of the whole society, when bovine milk is being introduced to them and being replaced by breast-milk.

Since TET residues may also be found in additional food sources, such as eggs and meat, it is possible that a societal group may receive residues of this drug type at a level greater than the defined ADI if several food sources are contaminated with tetracycline residues. A risk assessment study on TET residues through milk consumption carried out by the Environmental Protection Agency revealed that the estimated daily intake of oxytetracycline residues for Americans was 9.6 μg/person/day, which accounted for a low risk [32]. In Yugoslavia, a similar study showed that the hazards associated with tetracycline residue intake via milk was negligible [29].

It should be mentioned that in Iran, the average consumption of milk is lower than the recommended daily allowance (RDA) of two servings (or 500 ml) per day [23]. Therefore, increases in milk and dairy product intake at the advice of nutritionists can increase the risk of TET uptake through the food chain. Until recently, no national or administrative program has been introduced to monitor the presence of drug residues in milk in Iran. The results of the present study can be informative for the safety authorities to instigate new policies to restrict the present potential risk.

## Conclusion

In the present study, the average concentration of total TETs in milk was determined as 2.5 times greater than the maximum residue limit (MRL) set by codex and oxytetracyclin had the highest share in this contamination. Daily exposure to Tetracycline residues intake through milk considering long term and short term milk consumption were estimated to range from 58–62 μg and 0–99.3 μg per person.

## References

[1] Kapusnik-Uner JE, Sande MA, Chambers HF (1996). Tetracyclines, chloramphenicol, erythromycin and miscellaneous antibacterial agents. The Pharmacological Basis of Therapeutics.

[2] Furusawa F (1999). Rapid liquid chromatographic determination of oxytetracycline in milk. J Chromato A.

[3] Botsoglou NA, Fletuvris DJ (2001). Drug residue in food; Pharmacology,Food Safety, and Analysis. Chapter 3.

[4] Elmund GK, Morrison SM, Grant DW, Nevins MP (1971). Role of excreted chlorotetracycline in modifying the decomposition process in feedlot waste. Bulletin of Environmental Contamination and Toxicology.

[5] Fritz JW, Zuo Y (2007). Simultaneous determination of tetracycline, oxytetracycline, and 4-epitetracycline in milk by high-performance liquid chromatography. Food Chemistry.

[6] Kathleen P. Martindale: The Complete Drug Reference. 32th ed, 1 Chapter Antibacterials, UK, London: The Pharmaceutical press. 115–132.

[7] Olatoye, IO, Ehinmowo, AA. Oxytetracycline residues in edible tissues of cattle slaughtered in Akure, Nigeria. Nigerian. Nigerian Veterinary Journal. 2010;31(2):93-103.

[8] Codexalimentarius. Maximum Residue Limits for Veterinary Drugs in Foods. Updated as at the 35th Session of the Codex Alimentarius Commission 2012, 1–40. http://www.codexalimentarius.net/vetdrugs/data/vetdrugs/index.html?lang=en.

[9] European Commission. On pharmacologically active substances and their classification regarding maximum residue limits in foodstuffs of animal origin. Commission Regulation (EU). No 37/2010 of 22 December 2009, 1–76. http://ec.europa.eu/health/files/eudralex/vol-5/reg_2010_37/reg_2010_37_en.pdf.

[10] Applegren L, Arnold D, Boisseau J, Boobis A, Ellis R, Livingston R (1999). Evaluation of certain veterinary drug residues in food. Fiftieth report of the Joint FAO/WHO Expert Committee on Food Additives.

[11] US-Food and Drug Administration (FDA): Tolerance for residues of new animal drugs in food. Code of Federal Regulations 2012. http://www.accessdata.fda.gov/scripts/cdrh/cfdocs/cfCFR/CFRSearch.cfm?CFRPart=556&showFR=1

[12] Movassagh MH, Karami AR (2010). Determination of antibiotic residues in bovine milk in Tabriz. Iran. Global Veterinaria..

[13] Sani AM, Nikpooyan H, Moshiri R (2010). Aflatoxin M 1 contamination and antibiotic residue in milk in Khorasan province, Iran. Food and Chemical Toxicology..

[14] Movassagh MH (2011). Study of antibiotics residues in Cow Raw milk by Copan milk test in parsabad region, Ardabil province, Iran. Annals Biol Res..

[15] Movassagh MH (2012). Detection of antibiotics residues in cow raw milk in Bostanabad Region, Iran. Res Opin Anim Vet Sci..

[16] Joint FAO/WHO Expert Committee (JECFA) (2012). On Dietary Exposure Assessment Methodologies for Residues of Veterinary Drugs.

[17] Aalipour F, Maryam M, Jalali M (2014). Determination of antibiotic consumption index for animal originated foods produced in animal husbandry in Iran, 2010. J Env Health Sci Eng.

[18] Aalipour F, Mirlohi M, Jalali M (2013). Prevalence of contamination with antibiotic residues in commercial milk as affected by the season and different thermal processing. Int J Env Health Eng.

[19] Eliangiringa K, Chambuso M, Kitwala J (2008). Analysis of residual oxytetracycline in fresh milk using polymer reversed-phase column. Food Chem.

[20] Cinquina AL, Longo F, Anastasi G, Giannetti L, Cozzani R (2003). Validation of a high-performance liquid chromatography method for the determination of oxytetracycline, tetracycline, chlortetracycline and doxycycline in bovine milk and muscle. J Chromato A.

[21] Bratinova S, Raffael B, Simoneau C: Guidelines for performance criteria and validation procedures of analytical methods used in controls of food contact materials. EUR 24105 EN 2009, 1st ed. 1–74. http://www.irna.ir/en/News/2716240/Social/Iran%E2%80%99s_per_capita_milk_consumption.

[22] Azadbakht L, Mirmiran P, Esmaillzadeh A, Azizi F (2005). Dairy consumption is inversely associated with the prevalence of the metabolic syndrome in Tehranian adults. The Am J Clin Nut.

[23] Rafraf M, Bazyun B (2011). Food Habits Related To Osteoporosis in Women in Iran. Health promotion perspectives.

[24] Ramezani Tehrani F, Moslehi N, Asghari G, Gholami R, Mirmiran P, Azizi F (2013). Intake of dairy products, calcium, magnesium, and phosphorus in childhood and age at menarche in the Tehran lipid and glucose study. PloS one.

[25] Rassouli A, Abdolmaleki Z, Kamkar A, Shams GHR (2010). A cross- sectional study on oxytetracyclin and tetracyclin residues in pasteurized milk supplied in tehran by an HPLC method. International J Vet Res.

[26] Abbasi M, Mesgari B, Hossein A, Masoud N, Ashraf-o S, Nemati M (2011). Simultaneous Determination of Tetracyclines Residues in Bovine Milk Samples by Solid Phase Extraction and HPLC-FL Method. Ad Pharmac Bulletin.

[27] 28- Abebew DS: Detertion and determination of oxytetracycline and penicillin g antibiotic reidue levels in bovine bulk milk from Debrezeit and Nazareth dariy farms. Proceedings of the 1 International Technology, Education and Environment Conference, African Society for Scientific Research 2008. http://www.hrmars.com/admin/pics/241.pdf

[28] Vragović N, Bažulić D, Njari B (2011). Risk assessment of streptomycin and tetracycline residues in meat and milk on Croatian market. Food Chem Toxicol.

[29] Grădinaru AC, Popescu O, Solcan G (2011). Antibiotic residues in milk from Moldavia. Romania. HVM Bioflux.

[30] Molina MP, Althaus RL, Balasch S, Torres A, Peris C, Fernandez N (2003). Evaluation of Screening Test for Detection of Antimicrobial Residues in Ewe Milk. J Dairy Sci.

[31] Vragovic N, Bazulic D, Jakupovic E, Zdolec N (2012). Dietary exposure assessment of streptomycin and tetracycline in food of animal origin on the Croatian market. Food Addit Contam: Part B.

